# Clinical Factors, Preventive Behaviours and Temporal Outcomes Associated with COVID-19 Infection in Health Professionals at a Spanish Hospital

**DOI:** 10.3390/ijerph17124305

**Published:** 2020-06-16

**Authors:** Mario Rivera-Izquierdo, María del Carmen Valero-Ubierna, Silvia Martínez-Diz, Miguel Ángel Fernández-García, Divina Tatiana Martín-Romero, Francisco Maldonado-Rodríguez, María Rosa Sánchez-Pérez, Luis Miguel Martín-delosReyes, Virginia Martínez-Ruiz, Pablo Lardelli-Claret, Eladio Jiménez-Mejías

**Affiliations:** 1 Service of Preventive Medicine and Public Health, Hospital Universitario Clínico San Cecilio, 18016 Granada, Spain; mario.rivera.sspa@juntadeandalucia.es (M.R.-I.); mariac.valero.sspa@juntadeandalucia.es (M.d.C.V.-U.); silvia.martinez.sspa@juntadeandalucia.es (S.M.-D.); mangel.fernandez.garcia.sspa@juntadeandalucia.es (M.Á.F.-G.); divinat.martin.sspa@juntadeandalucia.es (D.T.M.-R.); francisco.maldonado.sspa@juntadeandalucia.es (F.M.-R.); 2 Department of Preventive Medicine and Public Health, University of Granada, 18016 Granada, Spain; luismiguelmr@ugr.es (L.M.M.-d.); lardelli@ugr.es (P.L.-C.); eladiojimenez@ugr.es (E.J.-M.); 3 Instituto de investigación biosanitaria de Granada, ibs.GRANADA, 18012 Granada, Spain; 4 Chair of Teaching and Research in Family Medicine SEMERGEN-UGR, University of Granada, 18016 Granada, Spain; mrsanchezperez@gmail.com; 5 Consorcio de Investigación Biomédica en Red (CIBER) de Epidemiología y Salud Pública de España, CIBERESP, 28029 Madrid, Spain

**Keywords:** follow-up, healthcare, professionals, COVID, SARS-CoV-2, PCR, negativization

## Abstract

The novel coronavirus disease (COVID-19) outbreak has quickly spread around the world, with Spain being one of the most severely affected countries. Healthcare professionals are an important risk group given their exposure. The aims of this study were to determine the prevalence of symptoms, main concerns as patients, preventive behaviours of healthcare professionals, and the different temporal outcomes associated with the negativization of PCR results. A total of 238 professionals were analysed and follow-up was conducted from 11 March to 21 April 2020 through clinical records, in-depth surveys, and telephone interviews. Symptoms, concerns, and preventive measures were documented, and temporal outcomes (start and end of symptoms, first positive PCR, and negativization of PCR) were analysed through survival analyses. A high prevalence of gastrointestinal symptoms (especially in women and older professionals), fever, cough, and fatigue were reported. The main concern was contagion in the work and home environment. Professionals (especially men) reported low use of face masks before the pandemic. Our analysis indicates that the median times for the negativization of PCR testing to confirm the resolution of infection is 15 days after the end of symptoms, or 25 days after the first positive PCR test. Our results suggest that these times are longer for women and for professionals aged ≥55 years, therefore follow-up strategies should be optimized in light of both variables. This is the first study we are aware of to report factors associated with the time to negativization of PCR results. We present the first rigorous estimates of time outcomes and hope that these data can be valuable to continue feeding the prediction models that are currently being developed. Similar studies are required to corroborate our results.

## 1. Introduction

Transmission of a new pneumonia-producing coronavirus was detected by the Wuhan Municipal Health and Sanitation Commission (Hubei Province, China) on 31 December 2019 [1], and the virus was identified by gene sequencing analysis of samples from the lower respiratory tract [2,3]. It was decided to name the infective agent severe acute respiratory syndrome coronavirus type 2 (SARS-CoV-2), and the disease COVID-19.

Following rapid transmission of the virus in China, many countries reported their first cases of COVID-19 pneumonia [4,5,6,7]. Since the World Health Organization (WHO) declared a pandemic on 11 March 2020, international efforts have been made to describe the clinical characteristics of patients with COVID-19 [8,9]. Spain is one of the most severely affected countries, with 208,389 reported cases and 21,717 reported deaths as of 22 April 2020 [10].

Research to date has noted that COVID-19 shows variable clinical characteristics [10,11,12]. Although several descriptive studies of hospitalized patients have appeared, no studies we are aware of thus far have provided a detailed report of the symptoms in health professionals. Developing appropriate follow-up strategies for these professionals seems essential to mitigate the negative effects of large numbers of professionals on sick leave at a time of high demand, while avoiding unnecessary risk exposures. In this connection, the main concerns among these professionals after receiving a diagnosis based on a positive nucleic acid real-time polymerase chain reaction (PCR) test result have not been analysed yet are potentially important for efforts to alleviate psychological effects, stress and suffering. In addition, preventive behaviours prior to the pandemic should be documented because this information will serve in the future to analyse whether COVID-19 has improved their awareness of such measures or led to changes in their professional practice.

A key requisite for appropriate and effective follow-up strategies is reliable data on the time that elapses to a first negative PCR test result, both after the first positive PCR result and after the end of the symptoms. The average time from symptom onset to recovery is considered to be 2 weeks for mild illness and 3–6 weeks for severe or critical illness in the general population [11], but the corresponding periods for health professionals are unknown, and few studies have reported specific analyses. Early follow-up with PCR testing for health professionals with a laboratory-confirmed diagnosis, on the one hand, may involve unnecessary risk exposure for other professionals and patients, inconvenience and resource expenses. On the other hand, delaying follow-up PCR testing may lead to prolonged sick leave and isolation at a time when health care demands are high.

The aims of the present study were to determine the prevalence of symptoms in health professionals infected by SARS-CoV-2 at a hospital in southern Spain (Hospital Universitario Clínico San Cecilio, Granada), to identify their preventive behaviours before the pandemic and main concerns as patients, and to report different temporal outcomes associated with the start and end of symptoms and the results of PCR testing.

## 2. Materials and Methods

We analysed a consecutive case series comprising all 76 symptomatic health professionals (total of professionals working in the hospital: 3600) who were seen at the Preventive Medicine Service at our hospital and who had a positive PCR test result for SARS-CoV-2 from 11 March 2020 to 13 April 2020. None of the persons we approached to request participation in the study declined. No participant was excluded due to missing data or other reasons. In our hospital, during the frame of this study, the frequency of PCR testing of laboratory-confirmed healthcare professionals was weekly and only after the resolution of symptoms. In the first visit, baseline information was obtained regarding sex, age, hospital service where they were employed, profession, previous diseases, symptoms, their main concerns (as an open question), and their preventive behaviours before the pandemic, such as hand hygiene and use of masks for themselves and for their patients. Their clinical records were reviewed and telephone interviews were conducted later (up to 21 April 2020) to obtain information regarding date of risk contact (if any), date of the start and end of symptoms, date of their first positive PCR result, date of their first negative PCR, and whether they needed hospitalization.

### 2.1. Analysis

The distribution of all baseline variables was obtained for the whole sample and for subsamples stratified by sex, age and hospitalization. Kaplan–Meier survival estimates for the whole sample and subsamples stratified by sex and age (less than 54 years vs. 55 years or more) were obtained for the following times to outcome or censoring (if any): from risk contact to start of symptoms, from start of symptoms to end of symptoms, from start of symptoms to first positive PCR result, from start of symptoms to first negative PCR result, from first positive PCR result to first negative PCR result, and from end of symptoms to first negative PCR result. The corresponding survival curves and the median and interquartile range (IQR) were obtained for each time interval. A Cox regression model was fitted for each outcome to obtain *p* values for their associations with sex and age. Stata 15 software was used for all analyses (StataCorp. 2017. Stata Statistical Software: Release 15. College Station, TX, USA: StataCorp LLC).

### 2.2. Ethical Considerations

This study was approved by the Provincial Ethics Committee (CEIM/CEI Provincial de Granada, Granada, Spain) under the designation COVID_19_Profesionales, code 0863-N-20. All health professionals asked to participate in the research voluntarily agreed to sign an informed consent form. The database was anonymized and all identifying variables were removed for analysis.

## 3. Results

Table 1 shows the distribution of baseline variable values in the whole sample and subsamples stratified by age and sex. The average age was 45.8 (sd: 11.4). Differences between sexes were identified for choking, nausea, and diarrhoea (more frequent in women). A total of 11 professionals (14.5%) required hospitalization. This proportion seemed slightly higher in males (17.4%) than females (13.2%) and higher in those older than 54 years (21.7%) compared to younger patients (11.3%), although none of these differences reached statistical significance. The average duration of hospitalization was 4.1 days. None of the patients required intensive care.

**Figure 1:**
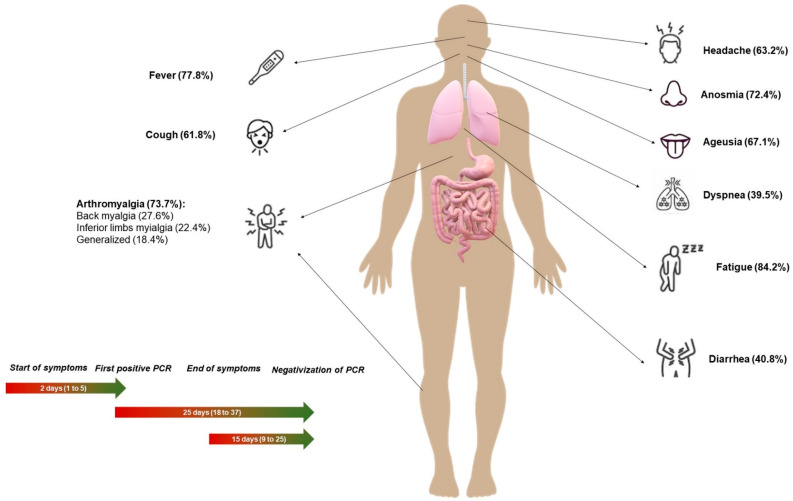
Most frequent symptoms and most relevant temporal outcomes in healthcare professionals with COVID-19.

A brief description of the main symptoms and most important time outcomes can be consulted in Figure 1.

The frequencies of preventive behaviours in health professionals before the COVID-19 pandemic are presented in Table 2. These frequencies tended to be higher in females, especially for self-use of masks. Table 2 also shows health professionals’ main concerns as patients. Thematic analysis of all open answers disclosed 4 groups of main concerns: spreading the disease in the environment, clinical deterioration and sequelae, psychological effects and loneliness due to confinement, and concerns about returning to work.

**Figure 2:**
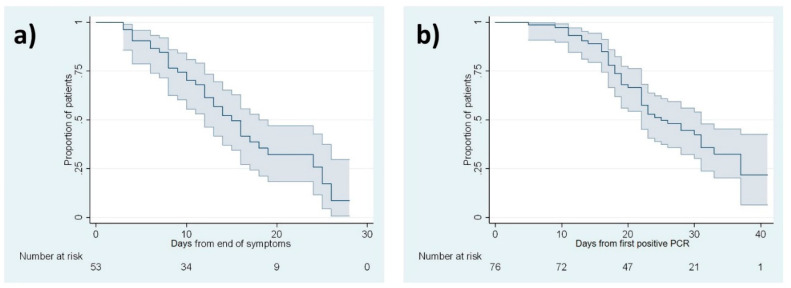
Kaplan–Meier survival analysis curves for negative PCR outcome. (**a**) Days from end of symptoms. (**b**) Days from first positive PCR. Shaded area: 95% confidence interval.

Table 3 shows the median times and IQR for each outcome: 2 days from symptom onset to first positive PCR result, 4 days from date of risk contact to symptom onset, 14 days from symptom onset to end of symptoms, 15 days from end of symptoms to negative PCR results (Figure 2a), 25 days from first positive PCR to first negative PCR (Figure 2b), and 31 days from symptom onset to first negative PCR. Times to the end of symptoms and negativization of PCR test results were longer in females and in patients older than 54 years. 

The estimation of the upper limit of the IQR (p75) could not be estimated for some outcomes (for example, a PCR negative result), because more than 25% of professionals had not yet developed them when the follow-up ended (censured data).

## 4. Discussion

Our series showed that only 26.3% of the infected professionals reported a perceived risk contact with patients, and most of the risk exposures were attributable to community or other professionals. The factors responsible of the contagion between professionals might also be analysed considering the starting date of confinement in Spain (15 March 2020).

### 4.1. Symptoms

The symptoms found in healthcare workers at our hospital contrast with information in a recent systematic review in China [13]. Although the frequency of fever (77.6% and 85.65%, respectively) and cough (61.8% and 65.7%, respectively) were similar, several other symptoms differed substantially. Fatigue (84.2% and 42.4%) and dyspnoea (39.5% and 21.4%) were more frequent in our case series, and other symptoms were surprisingly prevalent (ageusia 67.1%, anosmia 72.4%, headache 63.2%, arthromyalgia 73.7%). An earlier European study of 417 confirmed patients found high frequencies of olfactory (85.6%) and gustatory dysfunctions (88.0%) [14]. The frequencies in the present study are generally higher than in the ECDC reports [12]. These differences may be attributable to characteristics of our sample of patients, since symptomatic health professionals were presumably more likely to request PCR tests.

The prevalence of gastrointestinal symptoms reported by health professionals at our hospital was striking (diarrhoea, 40.8%, nausea, 22.4%; abdominal pain, 27.6%), and was higher than the figures reported by Assiri et al. [15] (diarrhea 26%, abdominal pain 17.0%) and in another study in Spain (diarrhoea 14.0%) [10] It was recently reported that ACE2 expression by enterocytes may influence the appearance of digestive tract manifestations [16]; this factor supports the biological plausibility of gastrointestinal symptoms in our patients.

In this connection, gastrointestinal and upper respiratory tract symptoms such as rhinorrhoea, sneezing, and sore throat are especially frequent in COVID-19 compared to other coronavirus diseases such as MERS or SARS [17].

Differences between sexes appeared for the frequency of nausea, diarrhoea and choking, which were reported more frequently by women. It was difficult to stratify our analysis according to the need for hospitalization because of the small number of participants who required hospitalization (only 14.5% of the sample). However, gastrointestinal symptoms and dyspnoea were more frequent in patients who needed hospitalization. These symptoms should be explored in future studies as possible predictors of the need for hospital care. The most notable age-related difference was the higher frequency of abdominal and rib pain and gastrointestinal symptoms in participants aged ≥55 years.

The variety of symptoms reported by our participants supports earlier evidence of the variability in clinical presentations of COVID-19. These differences in prevalence have several interpretations. First, our sample included health professionals of working age who were previously healthy, most of whom had no previous pathologies. In contrast, most studies published to date are based on data from hospitalized patients, many of whom had comorbidities or were older. Symptoms can be expected to differ between these two populations. In addition, our data are from structured and in-depth interviews with each participant, whereas data from medical records may be biased by the need for prompt (possibly rushed) entry and variability of clinical practices. Nonetheless, our data should be analysed with caution given the small sample size and the specific characteristics of our patient population. Another possible explanation for the differences in symptom frequencies across studies may lie in the base population of our study. Spanish health professionals may have perceptions that differ from those in other countries, or the virus may affect Spanish hospital healthcare staff differently due to as yet unidentified environmental, cultural or genetic factors. Despite these caveats, our data are potentially useful to identify healthcare professionals affected by COVID-19 in our setting.

### 4.2. Preventive Behaviours before the Pandemic

Health professionals’ self-reports generally suggested a reasonably appropriate level of hand hygiene in the workplace prior to the COVID-19 pandemic: 82.9% reported adequate hygiene, and the average self-score was 7.4 out of 10. However, the low rates of face mask use by healthcare staff (21.1%) and by patients (15.8%) were alarming.

Although it has been suggested that correct hand hygiene practices are not widely followed by health professionals (46.3% rate of adherence) [18], our results suggest that professionals at our hospital are more aware of preventive hand hygiene measures than the correct use of masks. Nevertheless, self-use of masks when respiratory symptoms (coughing, sneezing) are present, and the use of masks from the moment of first contact for patients with the same symptoms have been classically considered universal or standard precautions [19]. For reasons that should be explored in future studies, the importance of appropriate mask use has not been well acknowledged, and health education programs before the pandemic were apparently ineffective, even though seasonal Influenza viruses and agents that cause potentially serious illness (such as Neisseria meningitidis and Haemophilus influenzae, among others), can be transmitted through cough droplets. Future research should analyse the impact of the COVID-19 pandemic on health professionals’ awareness and implementation of preventive measures.

### 4.3. Main Concerns

Interest has increased in recent weeks in analysing and reducing the psychological burden, stress, and anxiety in both the general population [20] and health professionals [21,22]. The main concern expressed by our participants was spreading the disease in the environment (44.7%), especially to family members and older people they had close contact with. The second most frequent concern was clinical deterioration or sequelae (35.5%). The psychological effects of confinement, such as loneliness, lack of verified information, and feelings of abandonment were also identified as an important factor (7.9%). Finally, concerns about returning to work (4.0%), either as soon as their symptoms abated or later, e.g., when their PCR test result became negative, was identified as another stress factor.

These concerns should be considered when developing effective follow-up strategies aimed at alleviating anxiety in health professionals during isolation.

### 4.4. Follow-up Strategy: Median Times to Negative PCR Results

The median times showed in our study between the start of symptoms and the first positive PCR test suggest that prompt action from symptoms on to laboratory confirmation may be a good early identification strategy.

The core point in the follow-up of health professionals lies in the timing of initial and subsequent PCR testing, currently considered the standard method of verifying disease transmission [10,23]. Current recommendations suggest returning to work after a negative PCR test result, as long as symptoms are absent. Some studies have suggested that the likelihood of transmission is very low 1 week after the start of symptoms, despite a positive PCR test [24]. However, until this information is verified and widely accepted, no risks should be taken when deciding when it is safe for hospital staff to return to work after sick leave. It is important to remark that PCR positivity may not necessarily suggest infectivity. Currently, the Centres for Disease Control and Prevention (CDC) have moved away from a test-based approach with removal of isolation to a symptom-based approach [25]. According to this statement, data on PCR positivity and implication of viable virus have been described in a small study. Positive culture was only observed within 8 days of symptom onset [24].

The recommended timing for follow-up PCR testing has varied markedly since the start of the pandemic, and between different centres and countries. However, survival analyses are needed to report this information reliably so that it can contribute usefully to the available scientific evidence. Our results suggest follow-up PCR testing should be done at a median interval of 15 days (IQR 9 to 25) after the end of symptoms and 25 days (IQR 18 to 37) after the first positive PCR test. These intervals are similar to the period of 14 days after the start of symptoms currently recommended by WHO [11].

This proposal is based on data from up to five consecutive, positive PCR tests in some patients at the initial stages of the pandemic, a testing frequency that exposed other hospital staff members to the risk of contagion and generated unnecessary discomfort and concern. However, delaying follow-up PCR testing while patients remain on sick leave may mean that fewer staff are available for work at a time of high demand. Therefore, in view of our results, we suggest that follow-up PCR testing should take into account the median times reported in this work: 15 days after the end of symptoms or 25 days after the first positive PCR test result. However, future strategies should consider other remaining issues. For instance, the median time for first positive PCR will vary depending on how healthcare professionals are tested (e.g., if they are tested when asymptomatic or only when symptomatic and how frequently they are tested when asymptomatic). Furthermore, deciding how many positive tests should be done to confirm PCR negativity and when healthcare professionals become noninfectious remain uncertain to date.

Nevertheless, our results show some variability in the time to negativization of the PCR test results. To the best of our knowledge, no studies to date have focused on the differences in this interval between different groups of patients, except a recent published study of 56 patients [26]. In this work, the authors found that virus shedding was up to 6 weeks after onset of symptoms (we found a median time of 31 days) and, agreeing with our results, a prolonged observation period was necessary for older patients. Our results show that almost all temporal outcomes were longer in women, especially for the time between the onset and end of symptoms and the time between symptom onset and the first negative PCR result. Participants aged ≥55 years also took longer to reach most of the temporal outcomes, although these results were presumably somewhat imprecise because of the small sample size. Our results suggest that the timing of follow-up PCR testing should consider age and sex in health professionals with a laboratory-confirmed diagnosis of COVID-19. However, more studies with larger sample sizes are required to corroborate these results. We presented exploratory analyses that require further research controlling for other factors.

### 4.5. Strengths and Limitations

The sample for this study consisted of only 76 healthcare professionals. Given that follow-up lasted several weeks, and the first confirmed case was reported on 12 March 2020, we were unable to collect a larger sample, but we decided to analyse the data and report our findings promptly in order to support efforts to optimize follow-up strategies. The results from our small, specific sample should be contrasted with data from other hospitals and countries. Furthermore, PCR testing frequency is important for the interpretation of results. In this study, positive healthcare professionals were tested weekly, only after resolution of symptoms. 

Only the prevalence of symptoms was considered in all steps of data collection in this survey, which was carried out at the time negative PCR results became available. It was therefore not possible to undertake a temporal analysis of the order of appearance of symptoms. Future studies should consider the possibility of comparing symptoms between patients with laboratory confirmation of the diagnosis and patients with suggestive symptoms but no laboratory confirmation. Finally, the study design (case series) and the presence of censured values in the survival analysis make it advisable to interpret our results with caution. Nonetheless, our findings help to fill important gaps in our ability to detect the disease promptly and provide appropriate follow-up protocols for healthcare professionals, and provide evidence that will, we hope, serve to ground future decisions regarding the temporal outcomes that can be optimally studied with epidemiological or statistical methods. In sum, we present the first rigorous estimates of time outcomes and hope that these data can be valuable to continue feeding the prediction models that are currently being developed.

## 5. Conclusions

The symptoms in SARS-CoV-2-positive healthcare professionals in our study were variable, with a high prevalence of gastrointestinal symptoms (especially in women and older professionals), fever, cough, and fatigue. The main self-reported concern was contagion in the workplace and family settings, followed by possible clinical repercussions. Participants reported a low use of face masks before the pandemic. Our analysis indicates that the median time to negativization of PCR is 15 days after the end of the symptoms, or 25 days after the first positive PCR test. These intervals may be longer for women and for health professionals aged ≥55 years; therefore, follow-up strategies should be optimized on the basis of both variables. These data might help inform how long healthcare professionals are expected to remain PCR-positive and may be helpful for testing strategies.

## Figures and Tables

**Table 1:** Distribution of baseline characteristics in the whole sample and according to sex and age, and proportion of hospitalizations in each category.

Variable	Total	Males	Females	<55 Years	≥55 Years	Hospitalized
	*n* (%)	*n* (%)	*n* (%)	*n* (%)	*n* (%)	*n* (%) ^1^
Total	76 (100)	23 (30.3)	53 (69.7)	53 (69.7)	23 (30.3)	11 (14.5)
**Professional group**						
Medical	24 (31.6)	13 (56.5)	11 (20.7)	20 (37.7)	4 (17.4)	5 (20.8)
Nursing	39 (51.3)	6 (26.1)	33 (62.3)	25 (47.2)	14 (60.9)	4 (10.3)
Others	13 (17.1)	4 (17.4)	9 (17.0)	8 (15.1)	5 (21.7)	2 (50.0)
**Professional category**						
Physicians	15 (19.7)	7 (30.4)	8 (15.1)	11 (20.8)	4 (17.4)	4 (26.7)
Doctors in training	8 (10.5)	5 (21.7)	3 (5.7)	8 (15.1)	0 (0.0)	1 (12.5)
Nurses	25 (32.9)	6 (26.1)	19 (35.9)	17 (32.1)	8 (34.8)	2 (8.0)
Nursing assistants	15 (19.7)	1 (4.4)	14 (26.4)	9 (16.9)	6 (26.1)	2 (13.3)
Others	13 (17.1)	4 (17.4)	9 (17.0)	8 (15.1)	5 (21.7)	2 (15.4)
**Hospital service**						
Clinical specialties	16 (21.1)	3 (13.0)	13 (24.5)	7 (13.2)	9 (39.1)	2 (12.5)
Surgical specialties	23 (30.3)	10 (43.5)	13 (24.5)	19 (35.9)	4 (17.4)	3 (13.0)
Intensive care units	8 (10.5)	3 (13.0)	5 (9.4)	5 (9.4)	3 (13.0)	2 (25.0)
Emergencies	7 (9.2)	1 (4.4)	6 (11.3)	5 (9.4)	2 (8.7)	1 (14.3)
COVID hospitalization wards	9 (11.8)	1 (4.4)	8 (15.1)	7 (13.2)	2 (8.7)	1 (11.1)
Other services	13 (17.1)	5 (21.7)	8 (15.1)	10 (18.9)	3 (13.0)	2 (15.4)
**Perceived risk contact before infection**						
Professional/Colleague at the hospital	32 (42.1)	10 (43.5)	22 (41.5)	25 (47.2)	7 (30.4)	4 (12.5)
Patients	20 (26.3)	7 (30.4)	13 (24.5)	13 (24.5)	7 (30.4)	4 (20.0)
Community contact	12 (15.8)	5 (21.7)	7 (13.2)	6 (11.3)	6 (26.1)	4 (33.3)
**Risk contact after infection**						
Reported cases in their setting after infection	39 (51.3)	10 (43.5)	29 (54.7)	25 (47.2)	14 (60.9)	8 (20.5)
Reported cohabiting cases in their setting after infection	29 (38.2)	8 (34.8)	21 (39.6)	19 (35.9)	10 (43.5)	8 (27.6)
**Previous diseases**						
Diabetes mellitus	5 (6.6)	1 (4.4)	4 (7.8)	1 (1.9)	4 (17.4) *	1 (20.0)
Hypertension	8 (10.5)	2 (8.7)	6 (11.3)	3 (5.7)	5 (21.7) *	2 (25.0)
Chronic lung disease	5 (6.6)	1 (4.4)	4 (7.6)	3 (5.7)	2 (8.7)	1 (20.0)
Allergic asthma	8 (10.5)	3 (13.0)	5 (9.4)	6 (11.3)	2 (8.7)	1 (12.5)
None	63 (82.9)	21 (91.3)	42 (79.2)	47 (88.7)	16 (69.6)	7 (11.1)
**Symptoms**						
Fever < 38 °C	59 (77.6)	21 (91.3)	38 (71.7)	41 (77.4)	18 (78.3)	9 (15.3)
Fever > 38 °C	34 (44.7)	10 (43.5)	24 (45.3)	24 (45.3)	10 (43.5)	8 (23.5)
Upper respiratory tract symptoms	67 (88.2)	20 (87.0)	47 (88.7)	47 (88.7)	20 (87.0)	10 (14.9)
Cough	47 (61.8)	11 (47.8)	36 (67.9)	30 (56.6)	17 (73.9)	7 (14.9)
Throat pain	24 (31.6)	4 (17.4)	20 (37.7)	17 (32.1)	7 (30.4)	1 (4.2)
Sore throat	34 (44.7)	9 (39.1)	25 (47.2)	27 (50.9)	7 (30.4)	3 (8.8)
Choking	15 (19.7)	0 (0.0)	15 (28.3) *	11 (20.8)	4 (17.4)	2 (13.3)
Mucosal dryness	43 (56.6)	11 (47.8)	32 (60.4)	27 (50.9)	16 (69.6)	8 (18.6)
Rhinorrhoea	36 (47.4)	8 (34.8)	28 (52.8)	25 (47.2)	11 (47.8)	5 (13.9)
Otalgia	3 (4.0)	1 (4.4)	2 (3.8)	3 (5.7)	0 (0.0)	0 (0.0)
Headache	48 (63.2)	11 (47.8)	37 (69.8)	33 (62.3)	15 (65.2)	8 (16.7)
Periocular pain	25 (32.9)	6 (26.1)	19 (35.9)	16 (30.2)	9 (39.1)	4 (16.0)
Ageusia	51 (67.1)	16 (69.6)	35 (66.0)	34 (64.2)	17 (73.9)	7 (13.7)
Anosmia	55 (72.4)	17 (73.9)	38 (71.7)	38 (71.7)	17 (73.9)	8 (14.5)
Gastrointestinal symptoms	45 (59.2)	9 (39.1) *	36 (67.9) *	27 (50.9)	18 (78.3) *	9 (20.0)
Nausea	17 (22.4)	0 (0.0)	17 (32.1) *	10 (18.9)	7 (30.4)	4 (23.5)
Vomiting	7 (9.2)	0 (0.0)	7 (13.2)	4 (7.6)	3 (13.0)	2 (28.6)
Diarrhoea	31 (40.8)	5 (21.7)	26 (49.1) *	21 (39.6)	10 (43.5)	6 (19.4)
Abdominal pain	21 (27.6)	5 (21.7)	16 (30.2)	11 (20.8)	10 (43.5) *	3 (14.3)
Loss of appetite	12 (15.8)	2 (8.7)	10 (18.8)	6 (11.3)	6 (26.1)	2 (16.6)
Fatigue	64 (84.2)	21 (91.3)	43 (81.1)	43 (81.1)	21 (91.3)	10 (15.6)
Arthromyalgia	56 (73.7)	17 (73.9)	39 (73.6)	39 (73.6)	17 (73.9)	8 (14.3)
Generalized AM	14 (18.4)	7 (30.4)	7 (13.2)	10 (18.9)	4 (17.4)	1 (7.1)
Upper limb myalgia	11 (14.5)	3 (13.0)	8 (15.1)	7 (13.2)	4 (17.4)	1 (9.1)
Lower limb myalgia	17 (22.4)	2 (8.7)	15 (28.3)	12 (22.6)	5 (21.7)	2 (11.8)
Back myalgia	21 (27.6)	7 (30.4)	14 (26.4)	17 (32.1)	4 (17.4)	4 (19.0)
Dyspnoea	30 (39.5)	9 (39.1)	21 (39.6)	21 (39.6)	9 (39.1)	8 (26.7)
Rib pain	24 (31.6)	4 (17.4)	20 (37.7)	13 (24.5)	11 (47.9) *	5 (20.8)
Dermatological symptoms	11 (14.5)	4 (17.4)	7 (13.2)	7 (13.2)	4 (17.4)	1 (9.1)

* *p* < 0.05. ^1^ In parentheses, the proportion of hospitalized professionals in each category of baseline variables.

**Table 2:** Preventive behaviours prior to the COVID-19 pandemic and main concerns of health professionals during SARS-CoV-2 infection.

Variable	Total	Males	Females	*p*-Value
**Previous preventive behaviours**				
Adequate hand hygiene (yes/no) (n, %)	63 (82.9)	18 (81.8)	45 (84.9)	0.740
Hand hygiene (self-score 1–10) (mean, sd)	7.4 (2.3)	7.0 (0.5)	7.6 (0.3)	0.354
Self-use of masks (n, %)	16 (21.1)	1 (4.55)	15 (28.3)	0.022
Use of masks for patients (n, %)	12 (15.8)	3 (14.3)	9 (17.0)	0.777
**Main concerns**				
Spreading the disease in the environment (n, %)	34 (44.7)	11 (47.8)	23 (43.4)	0.721
Clinical deterioration or sequelae (n, %)	27 (35.5)	9 (39.1)	18 (34.0)	0.665
Psychological effects and loneliness of confinement (n, %)	6 (7.9)	1 (4.4)	5 (9.4)	0.450
Concerns about returning to work (n, %)	3 (4.0)	1 (4.4)	2 (3.8)	0.906

**Table 3:** Median times for the main outcomes.

Time-to-Outcome Intervals	Total Sample				Stratified for Sex			Stratified for Age		
					Males	Females		Age <55 Years	Age ≥55 Years	
	*n* at Day 0	Outcomes	Censored	Median (IQR)	Median (IQR)	Median (IQR)	*p*-Value ^1^	Median (IQR)	Median (IQR)	*p*-Value ^1^
Date of perceived risk contact to Start of symptoms ^2^	39	39	0	4 (3, 7)	5 (3, 7)	4 (2, 7)	0.387	5 (3, 7)	4 (2, 5)	0.125
Start of symptoms to End of symptoms	76	55	21	14 (10, -)	13 (8, 17)	17 (11, -)	0.024	14 (10, 22)	19 (11, -)	0.129
Start of symptoms to First positive PCR ^3^	76	76	0	2 (1, 5)	2 (1, 4)	3 (1, 5)	0.081	2 (1, 4)	3 (1, 6)	0.784
Start of symptoms to Negative PCR	76	44	32	31 (21, -)	21 (18, -)	33 (25, -)	0.025	28 (10, 39)	33 (25, -)	0.207
First positive PCR to Negative PCR	76	44	32	25 (18, 37)	19 (16, -)	30 (22, 37)	0.076	22 (17, 37)	31 (23, -)	0.119
End of symptoms to Negative PCR ^4^	53	33	20	15 (9, 25)	10 (8, 16)	17 (12, 24)	0.322	13 (8, 24)	18 (14, 26)	0.522

The upper limit (p75%) of the IQR could not be obtained in some groups due to censoring. ^1^ *p*-values assess differences in the hazard ratio of each outcome across age and sex groups, obtained from the corresponding Cox regression model including age and sex as covariates. ^2^ For this outcome, 37 patients could not recall any risk contact. ^3^ We assigned a value of 0.5 days in 10 patients who reported that symptom onset and their first positive PCR result occurred on the same day. ^4^ Of 55 patients with a date for end of symptoms (not censored for this outcome), one had a negative PCR result 4 days before the date of end of symptoms, and another had a negative PCR result the same date as the end of symptoms; both patients were excluded from this analysis.
